# Association of aberrant resting heart rates with dementia risk across the life‐course by a longitudinal multilevel analysis

**DOI:** 10.1002/alz70860_098385

**Published:** 2025-12-23

**Authors:** Shakiru A Alaka, So‐Fong C Ngan, Christopher Chen, Newman Sze

**Affiliations:** ^1^ Brock University, St. Catharines, ON, Canada; ^2^ National University Health System, NUHS, Singapore, Singapore, Singapore; ^3^ Memory, Ageing and Cognition Centre, National University Health System, Singapore, Singapore; ^4^ Brock University, St Catharines, ON, Canada

## Abstract

**Background:**

The underlying causes of dementia often emerge decades before clinical symptoms appear, with risks accumulating throughout the life‐course. However, younger adults are always neglected in dementia research. Failing to address the risk of dementias in younger adults will most likely lead to a cascading effect on brain health that persists through mid‐life to late life. Resting heart rate (RHR), a key determinant of cardiovascular health and perfusion, remains insufficiently understood its life‐course relationship with Alzheimer's disease (AD).

**Methods:**

We analyzed data from the National Alzheimer's Coordinating Center (NACC) across three age groups: young adults (18‐50 years), mid‐life (51‐64 years), and older adults (65 years). We adopted multi‐level logistic regression analysis to examine the longitudinal association between RHR and dementia while controlling for potential confounders, and the E‐value approach was applied to estimate robustness of this effect.

**Results:**

After adjusting for potential confounders, in young adults, RHR < 60 bpm was associated with increased dementia risk. Among older adults, RHR > 100 bpm was linked to a higher risk of developing dementia (OR = 1.38, 95% CI: 1.09 – 2.11). The corresponding E‐values for both young and older adult groups were 1.63 and 1.26 respectively, indicating that unaccounted for confounding variables with small‐to‐moderate sized is required to account for an away effect.

**Conclusion:**

Both low and high RHR are significant risk factors for dementia in specific age groups. These findings highlight the importance of monitoring RHR as part of dementia prevention strategies.

